# Community health worker intervention to prevent incident type 2 diabetes: A cluster randomized trial

**DOI:** 10.1371/journal.pone.0361464

**Published:** 2026-10-30

**Authors:** Mark D. Schwartz, Jiyuan Hu, Scott E. Sherman, Radhika Gore, Natalie Levy, Michael Tanner, Michael Bergman, Nadia Islam

**Affiliations:** 1 Department of Population Health, NYU Grossman School of Medicine, New York, New York, United States of America; 2 Department of Medicine, VA New York Harbor Healthcare System, New York, New York, United States of America; 3 Department of Medicine, NYU Grossman School of Medicine, New York, New York, United States of America; Tabba Heart Institute, PAKISTAN

## Abstract

**Objective:**

Type 2 diabetes mellitus (T2DM) affects millions of Americans, and prediabetes significantly increases progression to T2DM. Community Health Workers (CHWs) may help prevent diabetes through behavioral interventions, but their effectiveness in real-world settings remains uncertain. We evaluated whether a CHW intervention prevents incident T2DM among adults with prediabetes.

**Research design and methods:**

Pragmatic, cluster-randomized controlled trial in primary care clinics at the Veterans Affairs NY Harbor Healthcare System (Manhattan) and Bellevue Hospital, New York City. The study included adults aged 18–75 years with HbA1c 5.7–6.4% and no prior diabetes or use of diabetes medications other than metformin. Primary care teams were cluster-randomized to a 12-month CHW program—health action plans, motivational interviewing, health education, and community referrals—versus usual care. The primary outcome was incident diabetes during up to 6 months’ follow-up after the 12-month intervention. Secondary outcomes were changes in HbA1c, weight, blood pressure, lipids, and health care utilization.

**Results:**

Incident diabetes occurred in 5.9% of intervention patients versus 4.3% of controls (risk difference 1.6%; 95% CI −3.68% to 4.10%). No significant differences were observed in HbA1c, weight, blood pressure, or lipids. The intervention group had more primary care encounters (77.6% vs 62.5%) and emergency department encounters (35.0% vs 29.1%).

**Conclusions:**

In this pragmatic trial, a 12-month CHW program did not reduce incident diabetes among adults with prediabetes but was associated with greater health care engagement (more primary care visits). More intensive or standardized CHW models should be tested to determine whether increased engagement translates into long-term metabolic benefit.

**Trial registration:**

The study protocol was registered at ClinicalTrials.gov (NCT03006666, 12/27/2016). https://clinicaltrials.gov/study/NCT03006666.

## Introduction

Type 2 diabetes, a preventable chronic disease affecting 37.3 million Americans, has a prediabetes prevalence of up to 96 million, increasing the risk of diabetes [1,2]. Prediabetes is associated with an annual progression rate to diabetes of approximately 5–10%, underscoring the need for scalable preventive interventions. Proven behavior change interventions for weight loss and physical activity can reduce diabetes incidence in patients with prediabetes [3–6].

Practices face challenges delivering these strategies, such as limited time for staff, unclear delegation of outreach tasks, and insufficient skills for motivational counseling to optimize diabetes prevention [7–9]. Community Health Workers (CHWs), who are frontline health professionals from patients’ communities, can overcome these barriers [10–12]. A systematic review of 22 studies across diverse settings found CHW interventions improved glycemic control and weight loss and were cost-effective [13]. CHWs conduct behavioral counseling, promote lifestyle change, and extend care beyond the clinic [14–21].

However, questions remain about CHWs’ effectiveness in real-world settings [18,19,22]. This paper presents primary results from a cluster-randomized, pragmatic trial of CHW integration into primary care (PC) to prevent type 2 diabetes in a large at-risk population.

## Methods

The study protocol was described in detail previously, is summarized below, and adheres to the CONSORT guidelines [23,24].

### Study design

This was a pragmatic, cluster-randomized, controlled trial to evaluate whether CHW coaching is effective in preventing type 2 diabetes among PC patients with prediabetes. PC teams (n = 14 clusters) were randomized 1:1 to intervention or control group (7 teams each), stratified by clinical site (Veterans Affairs vs Bellevue). Randomization was conducted by the study statistician using simple random allocation; allocation was not concealed given the pragmatic design. The three-year study was conducted with a staggered entry design across overlapping, twelve-month intervention waves to account for CHW caseload limitations.

### Setting

The study was conducted from 2018–2022 in primary care clinics at the Veterans Affairs NY Harbor Healthcare System (VA), and Bellevue Hospital (BH), safety-net hospitals in New York City.

VA had ten general PC teams, each with one to five PC clinicians (total of 24). BH had four PC teams, each with about eight PC clinicians (total of 32).

### Study population

Eligible patients were drawn from PC panels and identified through the hospital’s corporate data warehouses (CDW). Patients were eligible if they were aged 18–75 years, spoke English or Spanish, had at least one glycosylated hemoglobin (HbA1c) result in the prediabetic range (5.7–6.4%) in the two years prior to the start of the intervention (to capture recent prediabetes while minimizing misclassification from reversion to normoglycemia), and were enrolled in a PC panel. The main exclusion criterion was a prior diabetes diagnosis, defined as any ICD diagnostic code for diabetes during ambulatory encounters in the 2 years prior to the intervention, or any prescription of a diabetes medication other than metformin alone [25]. PC clinicians in the intervention group reviewed the list of their eligible patients and could exclude patients they deemed inappropriate (typically <10%, most commonly due to panel misattribution, competing illness, or uncertainty about prediabetes status).

Following the recruitment period for intervention patients in each wave, we randomly selected eligible patients from the control PC teams for inclusion in the trial (control patients). The ratio of enrolled intervention to control patients ranged from 1:2–1:2.5, with the ratio determined by the number of eligible patients in control teams at the conclusion of the outreach period. Neither the patients nor their PC clinician in the control group were aware of their allocation into the trial.

### Intervention and control conditions

To begin each 12-month wave of the study (3 at VA, 4 at BH), we randomly selected 250 eligible intervention group patients for outreach and paired them with one of four CHWs. CHWs were selected to reflect their communities, including bilingual English-Spanish CHWs at Bellevue and veteran CHWs at the VA, ensuring cultural concordance and trust. They received training in motivational interviewing and health coaching, were supervised through weekly case conferences, and were recruited for experience in community engagement and patient support.

We mailed patients a letter from their PC clinician about their diabetes risk, explained the study, and introduced them to their assigned CHW. CHWs called patients to describe the program and obtain verbal, informed consent. They continued outreach until their caseload was reached.

Each wave had a recruitment period, followed by a 6-month intensive phase of CHW contact and a 6-month maintenance phase. CHWs conducted baseline intake surveys and developed individualized health action plans (HAPs) focused on weight, diet, and physical activity, which were updated during the intervention and shared with primary care clinicians. In the intensive phase, CHWs had up to 6 months of weekly phone calls or in-person meetings in patients’ homes, communities, clinics, or research offices. This was reduced to monthly follow-up calls during the maintenance phase. Patients could contact their CHW for additional support.

The four core components of the CHW-based intervention are described here.

Setting Goals and Creating the HAP: The patient and CHW collaboratively drafted the HAP, which focused on weight loss, diet, and physical activity goals for diabetes prevention. The HAP also specified how often patients planned to engage with the CHW and intervention activities. The HAP, along with 3 and 6-month updates, was shared with the patient’s PC clinician.

Health Education: CHWs led tailored health education sessions on topics like health self-management, exercise, and healthy eating. They provided patients with educational materials about diabetes, exercise and nutrition to prevent diabetes, recipes, and patient logs, all written at an 8th-grade reading level in English or Spanish.

Complementing PC Clinician Visits: CHWs called or met patients before and after PC clinician visits to discuss diabetes prevention with the doctor or review their suggestions.

Referral to Hospital and Community-Based Resources: CHWs advised patients on diabetes prevention programs at their hospital or community and referred them to services. Both the VA and BH had weight management clinics. They also guided patients to community resources like farmers markets, organizations, and social services.

Control patients continued to receive usual care from their primary care teams and had access to existing clinical services and programs but received no CHW outreach or other components of the CHORD intervention.

### Outcome measures

The study’s primary outcome was type 2 diabetes incidence within 6 months after a 12-month intervention, totaling 18 months. Type 2 diabetes was defined as at least one diabetes diagnosis code (ICD-9 codes 250, 357.2, 362.0, or 366.41) during the study. Secondary outcomes included weight change (pounds and BMI), glycemic control (HbA1C), blood pressure, and cholesterol levels. All outcomes were derived from routine clinical measurements in the electronic medical record without protocol-driven intervals. Health service utilization, including PC visits, ED visits, and hospitalizations, was also extracted from the EMR.

### Covariate measures

From the EMR, we measured smoking status, calculated Framingham risk scores, and recorded clinical diagnoses with at least two diagnostic codes for conditions associated with incident type 2 diabetes: chronic kidney disease, MASLD, non-alcoholic steatohepatitis, heart failure, hepatitis C, hyperlipidemia, hypertension, hyperuricemia or gout, prediabetes, ischemic heart disease, metabolic syndrome, obesity, peripheral vascular disease, and stroke [26–28].

### Data collection

Data from clinician-approved patients meeting eligibility criteria were extracted and merged into a secure study database. This pragmatic, population-based trial used measurements from regular EMR visits to assess implementation fidelity [29].

### Statistical analysis

Continuous variables were summarized for all patients, stratified by intervention arms, as mean ± standard deviation (SD). Categorical variables were presented as numbers and frequencies. Baseline measurements were compared between the control and intervention groups using the Wilcoxon rank sum test for continuous variables and Chi-square or Fisher’s exact test for categorical variables. The primary outcome of diabetes incidence was compared using Chi-square tests. Secondary outcomes were compared using linear regression, negative binomial regression, and logistic regression for continuous, count, and binary outcomes, respectively, with adjustment for sex, age, and PC clinician identifier. Given the low number of incident diabetes events, additional covariate adjustment was limited to avoid model overfitting. Analyses were conducted for baseline clinical and diagnostic variables, post-intervention variables, and differences between baseline and post-intervention measurements. Analyses were conducted using R version 3.4.1, with statistical significance set at P < 0.05. Sample size was determined to provide at least 80% power to detect a difference in annual diabetes incidence hazard rates of 3.5% in the intervention group versus 5.4% in the control group at a 0.05 significance level, as detailed in the published protocol. The calculation assumed an intracluster correlation coefficient of 0.01.

The study was reviewed and approved by NYU Grossman School of Medicine (Reference Number: i16-00690), and the independent Boards of VA NY Harbor Healthcare System (MIRB 01609) and Bellevue Hospital Center (STUDY00000844).

## Results

### Study population

Fig 1 shows that 4,580 patients were eligible for the study. Ten PC teams at the VA and four at Bellevue were randomly allocated to intervention or control groups within each practice site. Of the 2,280 patients randomized to the intervention group, 646 were excluded by PCPs. We randomly selected 1,374 to account for the caseload capacity of the CHWs (~72 patients per CHW per wave) while maintaining adequate statistical power. 849 intervention patients were enrolled, of whom 728 completed the baseline survey and received allocated intervention. 1,781 control patients were randomly selected from 2,300 eligible patients from the control PC team.

**Fig 1:**
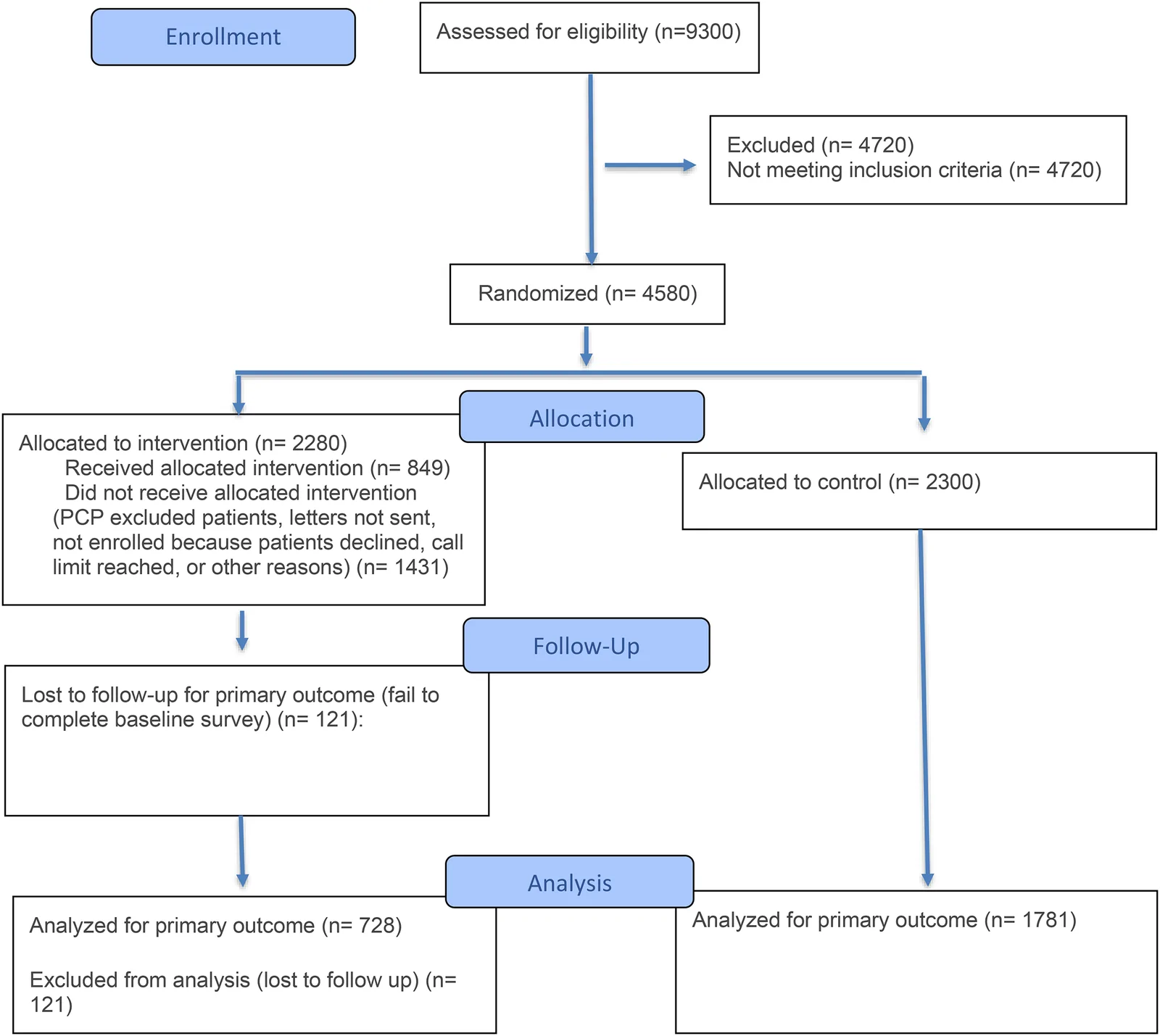
Study Enrollment Flow Diagram.

### Baseline characteristics (Table 1)

Thirty-nine percent of participants were female, with a mean age of 55.6 years. Many in the EMR had unknown racial and ethnic identities, but over a third identified as Black or African American, and at least 15.9% as Hispanic or Latino, with 13.8% speaking only Spanish. The control group was more likely to be female and slightly younger. Only intervention participants were asked to self-report ethnicity; ethnicity for control participants was obtained from the EHR and was frequently missing. Consequently, a greater proportion of intervention than control participants were identified as Hispanic or Latino. Other socioeconomic variables were similarly only measured for the intervention group.

**Table 1:** Baseline Patient Characteristics.

	Control	Intervention	Overall	P value*	Adjusted P Value**
**Sociodemographic Characteristics**	**N = 1781 (%)**	**N = 728 (%)**	**N = 2509 (%)**		
**Sex Female**	738 (41.4%)	238 (32.7%)	976 (38.9%)	<0.001	
**Age**	55.0 (12.2)	56.9 (12.0)	55.6 (12.2)	<0.001	
**Race**				0.016	
Asian	89 (5.0%)	26 (3.6%)	115 (4.6%)		
Black	601 (33.7%)	277 (38.0%)	878 (35.0%)		
White	345 (19.4%)	159 (21.8%)	504 (20.1%)		
Other/Unknown	746 (41.9%)	266 (36.5%)	1012 (40.3%)		
**Ethnicity**				0.003	
Hispanic or Latino	177 (9.9%)	222 (30.5%)	399 (15.9%)		
Not Hispanic or Latino	569 (31.9%)	502 (69.0%)	1071 (42.7%)		
Unknown	1035 (58.1%)	4 (0.5%)	1039 (41.4%)		
**Language**				0.524	
English	1378 (77.4%)	572 (78.6%)	1950 (77.7%)		
English and Spanish	148 (8.3%)	64 (8.8%)	212 (8.4%)		
Spanish	255 (14.3%)	92 (12.6%)	347 (13.8%)		
**Hospital**				<0.001	
Bellevue	1014 (56.9%)	353 (48.5%)	1367 (54.5%)		
VA	767 (43.1%)	375 (51.5%)	1142 (45.5%)		
Insurance***					
Insured	–	123 (15.5%)	–		
Self-pay/no insurance/unknown	–	615 (84.5%)	–		
Marital status***					
Married	–	213 (29.3%)	–		
Unmarried/Unknown	–	515 (70.75%)	–		
Education***					
Less than elementary	–	4 (0.5%)	–		
Elementary	–	37 (5.1%)	–		
Some high school	–	50 (6.9%)	–		
High school graduate	–	140 (19.2%)	–		
Some college or technical school	–	170 (23.4%)	–		
College graduate	–	117 (16.1%)	–		
Unknown	–	210 (28.8%)	–		
Employment status***					
Employed	–	258 (35.4%)	–		
Other****	–	506 (64.6%)	–		
**Clinical Characteristics**					
**Weight** pounds (SD)	183 (42.9)	187 (45.3)	184 (43.7)	0.05	0.15
**BMI** kg/m^2^ (SD)	29.4 (5.70)	29.4 (5.91)	29.4 (5.77)	0.99	0.26
**BMI category**				0.37	0.70
Healthy or underweight	292 (16.4%)	131 (18.0%)	423 (16.9%)		
Overweight	473 (26.6%)	250 (34.3%)	723 (28.8%)		
Obesity	533 (29.9%)	249 (34.2%)	782 (31.2%)		
Missing	483 (27.1%)	98 (13.5%)	581 (23.2%)		
**SBP** mm Hg (SD)	126 (16.3)	126 (15.9)	126 (16.2)	0.49	0.86
**DBP** mm Hg (SD)	76.9 (10.3)	77.2 (9.61)	77.0 (10.1)	0.60	0.97
**Lipids**					
Total Cholesterol mg/dl (SD)	185 (44.0)	184 (42.7)	185 (43.5)	0.91	0.92
HDL Cholesterol mg/dl (SD)	52.4 (14.4)	52.2 (14.4)	52.4 (14.4)	0.66	0.97
Triglycerides mg/dl (SD)	139 (88.9)	138 (87.0)	138 (88.2)	0.79	0.91
LDL Cholesterol mg/dl (SD)	105 (37.3)	106 (35.9)	106 (36.8)	0.67	0.37
**HbA1c** % (SD)	5.89 (0.246)	5.90 (0.275)	5.90 (0.255)	0.24	0.91
**Current smoking** # Yes	367 (20.6%)	180 (24.7%)	547 (21.8%)	0.03	0.52
**Framingham risk score** mean (SD)	0.058 (0.052)	0.068 (0.054)	0.062 (0.054)	<0.01	0.26
**Baseline Clinical Diagnoses**					
Chronic kidney disease	30 (1.7%)	13 (1.8%)	43 (1.7%)	0.99	0.36
Fatty liver disease	20 (1.1%)	9 (1.2%)	29 (1.2%)	0.97	0.97
Heart failure	30 (1.7%)	14 (1.9%)	44 (1.8%)	0.81	0.57
Hepatitis C	33 (1.9%)	17 (2.3%)	50 (2.0%)	0.53	0.73
Hyperlipidemia	258 (14.5%)	117 (16.1%)	375 (14.9%)	0.34	0.04
Hypertension	369 (20.7%)	179 (24.6%)	548 (21.8%)	0.04	0.51
Hyperuricemia or gout	0 (0.0%)	1 (0.1%)	1 (0.0%)	–	–
Impaired fasting glucose, Prediabetes	192 (10.8%)	64 (8.8%)	256 (10.2%)	0.16	0.03
Ischemic Heart Disease	79 (4.4%)	47 (6.5%)	126 (5.0%)	0.05	0.65
Metabolic Syndrome	2 (0.1%)	0 (0.0%)	2 (0.1%)	>0.99	>0.99
Obesity	142 (8.0%)	62 (8.5%)	204 (8.1%)	0.71	0.93
Peripheral vascular disease	19 (1.1%)	10 (1.4%)	29 (1.2%)	0.66	0.83
Stroke	9 (0.5%)	12 (1.6%)	21 (0.8%)	0.01	0.02

* P-value was obtained from Wilcoxon rank-sum test or Chi-square test.

** Adjusted p-value was obtained from linear or logistic regression or proportional odds model adjusting for Age, Sex and PC clinician identifier.

*** These variables were only available for Intervention Group subjects as the study did not directly engage with Control Group subjects. **** Employment categories harmonized across sites; ‘Other’ includes unemployed, retired, disabled, and other statuses.

Before intervention, the two groups were similar in weight, BMI, blood pressure, HbA1c, and lipids. The intervention group had a 20% higher smoking rate, contributing to a 17% higher Framingham risk score, but these differences were not statistically significant after adjusting for sex, age, and PC clinician identifier. The groups were similar in baseline comorbidities associated with incident diabetes. All patients had at least one prediabetes HbA1c level in the two years before enrollment, but the control group had a higher rate of prediabetes diagnosis codes (10.8% vs. 8.8%).

### Primary outcome results (Table 2)

At trial’s end, 43 (5.9%) patients in the Intervention group had incident diabetes, compared to 76 (4.3%) in the Control group, a non-significant risk difference of 1.6% (95% CI [−3.68%, 4.10%]).

**Table 2:** Primary and Secondary Clinical Outcomes.

	Control	Intervention	Overall	P value*	Adjusted p-value**
**Post intervention**	**(N = 1781)**	**(N = 728)**	**(N = 2509)**		
Diabetes onset N (%)	76 (4.3%)	43 (5.9%)	119 (4.7%)	0.10	0.28
HbA1c % (SD)	5.96 (0.583)	5.97 (0.718)	5.96 (0.633)	0.71	0.92
Weight pounds (SD)	180 (41.9)	184 (40.7)	181 (41.6)	0.02	0.28
BMI kg/m^2^ (SD)	29.4 (5.82)	29.3 (5.58)	29.3 (5.74)	0.71	0.93
Systolic blood pressure mm Hg (SD)	128 (15.8)	127 (15.3)	128 (15.7)	0.19	0.11
Diastolic blood pressure mm Hg (SD)	76.6 (10.2)	77.2 (10.2)	76.8 (10.2)	0.89	0.52
Total Cholesterol mg/dl (SD)	178 (40.8)	181 (42.0)	179 (41.2)	0.27	0.10
Triglycerides mg/dl (SD)	133 (88.6)	129 (77.7)	132 (84.9)	0.75	0.44
HDL cholesterol mg/dl (SD)	54.0 (16.0)	53.4 (16.3)	53.8 (16.1)	0.43	0.96
LDL cholesterol mg/dl (SD)	98.2 (34.0)	102 (34.7)	99.4 (34.3)	0.16	0.06
**Difference post intervention from baseline**					
HbA1c % (SD)	0.0436 (0.497)	0.0417 (0.693)	0.0429 (0.572)	0.32	0.78
Weight pounds (SD)	−0.117 (9.16)	0.361 (10.7)	0.0480 (9.73)	0.67	0.41
BMI (SD)	−0.107 (2.46)	−0.170 (2.47)	−0.129 (2.46)	0.79	0.68
Systolic blood pressure mm Hg (SD)	1.89 (16.1)	1.67 (16.2)	1.81 (16.1)	0.59	0.82
Diastolic blood pressure mm Hg (SD)	0.0281 (10.3)	0.756 (10.7)	0.282 (10.4)	0.40	0.22
Total Cholesterol mg/dl (SD)	−3.57 (31.6)	−3.02 (30.5)	−3.34 (31.1)	0.94	0.58
Triglycerides mg/dl (SD)	−5.17 (83.2)	−8.81 (65.8)	−6.57 (76.9)	0.57	0.47
HDL cholesterol mg/dl (SD)	0.729 (9.23)	0.347 (7.83)	0.582 (8.71)	0.53	0.77
LDL cholesterol mg/dl (SD)	−4.67 (29.1)	−4.97 (27.4)	−4.78 (28.4)	0.85	0.93

* P-value was obtained from Wilcoxon rank-sum test or Chi-square test.

** Adjusted p-value was obtained from negative binomial or logistic regression adjusting for Age, Sex and PC clinician identifier.

### Secondary outcome results

After adjusting for sex, age, and PC clinician identifier, the intervention and control groups had similar HbA1c, weight, BMI, blood pressure, and lipid levels at trial’s end. Changes from baseline to end of trial were similar between groups.

The intervention and control groups had few differences in the proportion of patients with most medical comorbidities at trial’s end. The intervention group had a higher rate of ischemic heart disease (10.6% vs. 6.4%, risk difference 4.2%, 95% CI [1.6%, 6.8%]). In unadjusted analyses, the intervention group had higher rates of hyperlipidemia (18.0% vs. 13.9%, difference 4.1%, 95% CI [0.75%, 7.4%]) and hypertension (32.8% vs. 27.8%, difference 5.0%, 95% CI [0.94%, 9.2%]) than the control group. There were no differences in chronic kidney disease, heart failure, metabolic syndrome, obesity, peripheral vascular disease, or stroke.

The outcomes remained unchanged during the 6-month intensive phase and the 6-month maintenance phase of the intervention, and when the outcome period was restricted to 12 months in each study wave.

### Health care utilization (Table 3)

During the trial, patients in the intervention group had more PC encounters (77.6% vs 62.5%, difference of 15.1%) and ED visits (35.0% vs 29.1%, difference of 5.9%) than those in the control group. Hospitalization rates and average hospitalizations per patient were low and similar between the two groups.

**Table 3:** Health Care Utilization during Study Period.

	Control	Intervention	Overall	P value*	Adjusted p value**
	**(N = 1781)**	**(N = 728)**	**(N = 2509)**		
Patients had primary care encounter/s N (%)	1114 (62.5%)	565 (77.6%)	1679 (66.9%)	<0.01	<0.01
Number of primary care encounters (SD)	2.33 (3.29)	2.87 (3.36)	2.48 (3.32)	<0.01	<0.01
Patients had Emergency Department encounter/s N (%)	519 (29.1%)	255 (35.0%)	774 (30.8%)	<0.01	0.31
Number of Emergency Department encounters (SD)	0.540 (1.23)	0.728 (1.57)	0.595 (1.34)	<0.01	<0.01
Patients had hospitalization/s N (%)	138 (7.7%)	74 (10.2%)	212 (8.4%)	0.06	0.81
Number of hospitalizations (SD)	0.115 (0.500)	0.168 (0.644)	0.130 (0.546)	0.05	0.35

* P-value was obtained from Wilcoxon rank-sum test or Chi-square test.

** Adjusted p-value was obtained from negative binomial or logistic regression adjusting for Age, Sex and PC clinician identifier.

## Discussion

This pragmatic cluster-randomized trial provides insights into CHWs’ role in preventing type 2 diabetes in real-world safety-net PC populations. Despite a strong theoretical framework and prior evidence, our findings reveal a more nuanced picture.

The CHORD study failed to show a reduction in type 2 diabetes incidence in the intervention group compared to the control group, contradicting prior research on lifestyle interventions for high-risk individuals [4,5]. Unlike prior efficacy trials, our pragmatic design, lower intervention intensity, and enrollment of patients already engaged in primary care likely attenuated observable effects. In our two safety-net clinics, the personalized support and education provided by CHWs may not have been intensive enough to reduce diabetes incidence [23]. Since the control group patients had higher baseline prediabetes diagnosis codes, their clinicians may have been more aware of this risk, making it harder to achieve a difference in incidence. The study’s 4.7% type 2 diabetes incidence was lower than the estimated 10% per year for those with prediabetes in a recent review, suggesting a lower-risk population and reduced power to detect differences [30].

Secondary outcomes, including weight, BMI, blood pressure, and lipid levels, were similar between the intervention and control groups, consistent with some real-world CHW studies showing limited metabolic change despite improved engagement [31,32]. Without differential weight loss, the lack of impact on diabetes incidence is unsurprising. The CHW intervention did not lead to substantial health status improvements compared to usual care. This may reflect baseline characteristics of a population already engaged in PC.

Additionally, the intervention might not have been sufficiently intensive or long to manifest significant changes in these clinical parameters, as described in our paper on implementation fidelity in this study [29^].^ In the implementation fidelity analysis, adherence to core components was high, with nearly 80.0% of intervention group patients having at least one successful encounter with their CHW, setting ≥ 1 goal, having ≥ 1 PC visit, and receiving ≥ 1 education session.

Notably, we observed more PC and ED visits in the intervention group, likely reflecting increased engagement driven by the CHW intervention, including greater detection and management of comorbid conditions [33,34]. Prior CHW studies have shown improved access to care and, in some settings, increased PC use, while broader continuity-of-care literature links sustained engagement with better chronic disease outcomes [35,36]. For example, the higher observed rates of ischemic heart disease may reflect increased diagnosis rather than true incidence. Other studies have found mixed impact of CHWs on rates of PC and ED visits and costs [31,32].

Limitations include baseline group differences, post-randomization exclusions, incomplete intervention uptake, and variability in CHW implementation. The lower-than-planned intervention intensity (mean ~5 encounters vs. expected weekly/monthly contacts) likely limited effectiveness. Differential exclusions may have introduced selection bias, potentially attenuating observed differences. Eligibility was based on an HbA1c in the prediabetes range within the preceding two years; some participants therefore may have reverted to normoglycemia or progressed to diabetes before enrollment. The algorithm used to exclude prevalent diabetes identified ambulatory diabetes diagnosis codes during the preceding two years; therefore, patients with an earlier diabetes diagnosis that was not recoded during that interval could have been misclassified as not having diabetes. Missing data and reliance on routine clinical measurements may have reduced sensitivity to detect differences. Strengths include the pragmatic, real-world design, large, diverse safety-net population, and integration within routine PC workflows, enhancing generalizability.

The CHORD study highlights the importance of exploring effective CHW strategies for chronic disease prevention. While it didn’t show significant diabetes reduction, increased healthcare utilization in the intervention group suggests CHWs may enhance patient engagement, potentially leading to long-term benefits for disease management and prevention.

## Supporting information

S1 File CONSORT 2025 checklist. (DOCX)
