# Supplementary material for: Community health worker intervention to prevent incident type 2 diabetes: A cluster randomized trial
Source: PLoS One. 2026 Oct 30;21(10):e0361464. doi: 10.1371/journal.pone.0361464 (PMC13722175; doi:10.1371/journal.pone.0361464)
Supplement: Supplementary file 2 [file pone.0361464.s003.docx]

**PLOS One: PONE-D-25-65348**

Community Health Worker Intervention to Prevent Incident Type 2 Diabetes

**Response to Editorial Review**

- NOTE: Please see our bulleted responses below each item as needed

**Journal requirements:**

When submitting your revision, we need you to address these additional requirements.

1. Please ensure that your manuscript meets PLOS ONE's style requirements, including those for file naming. The PLOS ONE style templates can be found at

- [https://journals.plos.org/plosone/s/file?id=wjVg/PLOSOne_formatting_sample_main_body.pdf](https://urldefense.com/v3/__https:/journals.plos.org/plosone/s/file?id=wjVg*PLOSOne_formatting_sample_main_body.pdf__;Lw!!Cs6gcNsejA!AvsyHOAvNeKyXL6vKa-XTXtJKG1Q2oa6lxhnuzesu7wRAy9dulE0AgYokqKXN1xEoFbhZiog8pzmw27sOX1fRN4_$) and
- [https://journals.plos.org/plosone/s/file?id=ba62/PLOSOne_formatting_sample_title_authors_affiliations.pdf](https://urldefense.com/v3/__https:/journals.plos.org/plosone/s/file?id=ba62*PLOSOne_formatting_sample_title_authors_affiliations.pdf__;Lw!!Cs6gcNsejA!AvsyHOAvNeKyXL6vKa-XTXtJKG1Q2oa6lxhnuzesu7wRAy9dulE0AgYokqKXN1xEoFbhZiog8pzmw27sOYZ_8TsE$)

2. Thank you for stating the following financial disclosure:

“This study was supported by a grant (R18DK110740) from the National Institute of Diabetes and Digestive and Kidney Diseases (NIDDK).”

Please state what role the funders took in the study.  If the funders had no role, please state: "**The funders had no role in study design, data collection and analysis, decision to publish, or preparation of the manuscript**."

If this statement is not correct you must amend it as needed. Please include this amended Role of Funder statement in your cover letter; we will change the online submission form on your behalf.

- This statement above is accurate, and we added this to our cover letter.

3. Thank you for stating the following in your manuscript:

“This study was supported by a grant (R18DK110740) from the National Institute of Diabetes and Digestive and Kidney Diseases (NIDDK). The funder monitored study progress but did not have a role in its design, implementation, analysis, or the preparation of this paper.  “

We note that you have provided funding information that is currently declared in your Funding Statement. However, funding information should not appear in the Acknowledgments section or other areas of your manuscript. We will only publish funding information present in the Funding Statement section of the online submission form. Please remove any funding-related text from the manuscript and let us know how you would like to update your Funding Statement.

- We removed this statement from the acknowledgments section.

4. Please provide a complete Data Availability Statement in the submission form, ensuring you include all necessary access information or a reason for why you are unable to make your data freely accessible. If your research concerns only data provided within your submission, please write "All data are in the manuscript and/or supporting information files" as your Data Availability Statement.

- We provided a complete data availability statement in the submission form.

**REVIEWERS' COMMENTS:**

**Comments to the Author**

1. Is the manuscript technically sound, and do the data support the conclusions?
The manuscript must describe a technically sound piece of scientific research with data that supports the conclusions. Experiments must have been conducted rigorously, with appropriate controls, replication, and sample sizes. The conclusions must be drawn appropriately based on the data presented.

Reviewer #1: Yes

Reviewer #2: Partly

Reviewer #3: Yes

2. Has the statistical analysis been performed appropriately and rigorously?

Reviewer #1: No

Reviewer #2: Yes

Reviewer #3: Yes

3. Have the authors made all data underlying the findings in their manuscript fully available?
The [PLOS Data policy](https://urldefense.com/v3/__http:/track.editorialmanager.com/CL0/http:*2F*2Fwww.plosone.org*2Fstatic*2Fpolicies.action*23sharing/1/010f019d09426359-dfa51069-96f6-4c73-a9bb-dc5b324b6ffd-000000/N0QQiyqf6VohS9jd5a6yDjnsXOxYX1OzQrQXHCiUhHE=255__;JSUlJSU!!Cs6gcNsejA!AvsyHOAvNeKyXL6vKa-XTXtJKG1Q2oa6lxhnuzesu7wRAy9dulE0AgYokqKXN1xEoFbhZiog8pzmw27sOSvxGQjz$) requires authors to make all data underlying the findings described in their manuscript fully available without restriction, with rare exception (please refer to the Data Availability Statement in the manuscript PDF file). The data should be provided as part of the manuscript or its supporting information or deposited to a public repository. For example, in addition to summary statistics, the data points behind means, medians and variance measures should be available. If there are restrictions on publicly sharing data—e.g. participant privacy or use of data from a third party—those must be specified.

Reviewer #1: Yes

Reviewer #2: Yes

Reviewer #3: No

4. Is the manuscript presented in an intelligible fashion and written in standard English?
PLOS ONE does not copyedit accepted manuscripts, so the language in submitted articles must be clear, correct, and unambiguous. Any typographical or grammatical errors should be corrected at revision, so please note any specific errors here.

Reviewer #1: Yes

Reviewer #2: Yes

Reviewer #3: Yes

**Review Comments to the Author**

**Reviewer #1**: The team conducted a pragmatic, cluster-randomized controlled trial to evaluate whether community health workers intervention can prevent incident T2DM among adults with prediabetes. The results from a 12-month CHW program don’t show significant differences in T2D incident between intervention and control groups.

1. Make sure to spell out the abbreviation for the first appearance. For example, what’s PC?

- We spelled out all abbreviations the first time they appear. PC is Primary Care.

2. Please clarify how control/intervention PC was determined

- Control versus intervention PC arms of the study was determined via a cluster randomization process, randomizing PC teams, stratified within the two clusters or hospital sites, Bellevue and VA.

3. Many exclusions occurred after randomization, which may lead to biased effect estimates.

- Most of the exclusions (4720) occurred before randomization, due to patients not meeting inclusion criteria. Among the 2280 patients cared for by intervention group teams, 1431 did not receive the intervention. Most of these were administrative exclusions due to the patient having left NYC, or the patient having no updated contact information.
- We addressed this issue in the limitation section of the discussion.

4. Sample characteristics at baseline were significantly different but they were not adjusted in the model, which would lead to confounding effect.

- In the analysis of primary outcome, we have conducted both univariate and multivariate approaches. Specifically, we used Chi-square test as univariate approach to compare the rate of diabetes between groups. We also used logistic regression model with adjustment of sex, age, and PC clinician identifier to account for clustering by clinician. No other covariates are further included in the model due to the low incident rate of diabetes.

5. Please clarify the cluster-randomization”. For example, how many clusters per arm? What’s cluster size and so on.

- There were 14 PC teams (clusters) in total, with10 at VA (16,000 patients) and 4 at Bellevue (26,000 patients). We randomly allocated teams to intervention versus control group, 7 PC teams for each, stratified by the two clinical sites. VA PC teams had from 1 to 5 PCPs (total = 24), and Bellevue PC teams had an average of 8 PCPs (total = 32).

**Reviewer #2**: First, I congratulate the authors on this manuscript, which addresses a relevant topic: lifestyle interventions delivered by community health workers hold promise for preventing type 2 diabetes mellitus. The following considerations are offered to further strengthen the work.

Introduction
1. The introduction is clear and direct; however, it would benefit from a more detailed description of diabetes mellitus and its implications, along with greater emphasis on the urgency of intervention in prediabetes.

- We have added this brief description to the introduction.

2. The systematic review in the second paragraph requires additional details, including the number of included studies, the total number of participants, and the geographic regions covered.

- We clarified these details in this paragraph.

3. I recommend rewriting the final sentence of the second paragraph for clarity and supporting it with an appropriate reference.

- We revised and clarified this sentence.

Methods
4. Although the manuscript references a previously published protocol, it does not provide the registration number or full publication details.

- The details and full citation for our published protocol are now included.

Setting
5. A figure illustrating the content of this section could substantially enhance readers’ understanding of the study.

- We defer to the editor regarding additional figures in this paper since we are already at the maximum number.

Study Population
6. The inclusion criterion specifies participants with a history of prediabetes within the last two years; However, the rationale for selecting this timeframe is unclear, as biochemical parameters may change substantially over longer periods.

- We chose the two-year timeframe prior to study enrollment to balance two goals: to capture most patients with a glycohemoglobin result in the prediabetic range, and to avoid too long a period to limit the likelihood of results returning to normal before enrollment.

7. Regarding exclusion criteria, only a diagnosis of diabetes within the last two years is mentioned, despite diabetes being a chronic, incurable condition—individuals diagnosed earlier retain the diagnosis lifelong. Furthermore, the other exclusion criteria require clarification, including rephrasing for precision in the sentence: “PC clinicians in the intervention group reviewed the list of their eligible patients and could exclude any they determined not appropriate for the intervention.”

- Before each intervention wave, PCPs were allowed to exclude any patients they thought to be inappropriate for the study. This was uncommon, less than 10%, with the most common reasons being that PCP did not recognize the name from their panel, patient has other significant illness, or no known prediabetes diagnosis.
- We added these details to the methods section.

8. The manuscript lacks essential details on randomization, including who generated the random allocation sequence, the method and type of randomization employed, and any restrictions used. It also omits the mechanism for implementing the sequence (e.g., steps to conceal allocation until interventions were assigned). A dedicated section describing this study stage would improve transparency and reproducibility.

- The team statistician conducted simple randomization and was unblinded to the randomization due to the nature of pragmatic trials. There was no step to conceal allocation once the randomization was determined at the beginning of the trial.

Intervention and Control Conditions
9. The method of contact with participants varied; a subanalysis could assess whether this factor influenced final outcomes. Similarly, participants who sought additional contacts with health agents may have achieved different outcomes than those who received only the scheduled interactions. A figure could more clearly illustrate the intervention logistics. Furthermore, no information is provided regarding the care received (or withheld) from the control group.

- We appreciate this suggestion, but we do not have the granularity to associate contact frequency with outcome variables. Control group patients received usual care from their clinical teams, but no engagement by the CHWs in the intervention group.

Outcome Measures
10. Change in weight in pounds (and body mass index, BMI, kg/m2), glycemic control (HbA1C), blood pressure, and cholesterol levels were secondary outcomes considered, but it was not described how and how frequently these variables were assessed.

- All outcome variables were assessed from the medical records as recorded by the clinic teams and health systems in the normal course of caring for patients.

Statistical Analysis
11. The manuscript does not clarify whether normality was assessed for continuous variables. Although R software is mentioned for statistical analyses, the specific packages and version employed are not reported. Furthermore, no description of the sample size calculation is provided to justify statistical power.

- We used non-parametric methods, i.e., Kruskal-Wallis test to conduct group comparisons, so normality was not required. We used R version 3.4.1 for analysis. The sample size calculation was detailed in the protocol paper: Islam N, et al. BMC Public Health. 2018;18(1):1–11.

Results
Study Population
12. The manuscript provides no explanation of how the 1374 participants were selected or the rationale for this specific sample size.

- As described in our protocol paper, cited above, the sample size was determined to provide at least 80% power to detect an expected difference in annual hazard rates of time to DM incidence between two arms (3.5% per year in intervention vs. 5.4% per year in control), at a 0.05 significance level. We randomly selected 1374 of the eligible intervention patients to account for the caseload capacity of the CHWs, assuming a caseload of 72 patients per full-time CHW per intervention wave.

Discussion
13. The first paragraph states that this study did not demonstrate a reduction in diabetes incidence in the intervention group, unlike prior reports. It would be valuable to highlight methodological and intervention differences that may account for this finding.

- Potential explanations for this finding are described in the discussion section.

14. The manuscript omits a discussion of secondary outcomes. Addressing findings from similar recent studies and comparing them to the present results would enrich the work.

- The secondary outcomes are discussed in the paragraph devoted to these in the discussion.

15. In the second paragraph, the statement “This may be a positive outcome, as increased engagement with healthcare providers is associated with better management of chronic conditions” lacks a reference. Regarding this statement, it is noteworthy that this study demonstrated a higher rate of comorbidity development (e.g., ischemic heart disease) in the intervention group—the very group that sought health services more frequently. Discussing this finding would also be valuable.

- We added this reference to support the assertion the reviewer highlighted:
- García-Villarino M, Martínez-Camblor P, García AV, Villa-Fernández E, Pérez-Fernández S, Lambert C, et al. Impact of general practitioner appointment frequency on disease management in type 2 diabetes mellitus patients. Prim Care Diabetes. 2025 Apr;19(2):165–172. doi:10.1016/j.pcd.2025.01.007.

16. In addition to outlining the study's limitations, highlighting its positive aspects is essential.

- We appreciate this comment and have added a discussion of the studies strengths to the discussion.

17. Finally, two key points regarding the study's limitations should be added: differences in baseline characteristics between the two groups and disparities in group sizes (intervention versus control).

- Thank you for noting the importance of these differences. We noted these in the limitations section.

**Reviewer #3**: This interesting paper describes a well-designed large pragmatic trial of a community health worker intervention for pre-diabetic patients. Although the study was ultimately a negative trial, the rigorous yet pragmatic methods for assessing this CHW intervention are an important contribution to the literature. Other strengths include large sample size and appropriate analytic methods. The authors note that the intensity of the intervention may not have been sufficient to demonstrate diabetes prevention; this insight could be explored more by incorporating additional results from the authors’ similarly well-designed manuscript assessing intervention fidelity.

- Thank you for this suggestion. We incorporated relevant findings from our paper on intervention fidelity.

Authors have not included data underlying findings in the manuscript or in a publicly accessible database, but authors describe that it can be made available without restrictions. However, they note that some data currently sit behind VA firewalls, and the VA typically tightly restricts data sharing. Per PLOS One guidance, the authors should be more explicit about the process for obtaining data and any restrictions on data sharing.

- We now clarify the process and restrictions for data sharing.

The manuscript is well-written and clear. The following minor edits would strengthen clarity:
- Spell out “Veterans Affairs New York” before using VA NY acronym in both abstract and manuscript body.
- On page 9, there is an extraneous parenthesis at the end of the first sentence.

- Thank you for noting these, both fixed.

Some additional suggestions that may strengthen this manuscript:
- Additional description of CHWs characteristics, hiring process, training, and supervision would be valuable. How were CHWs identified and hired? Were they selected for specific skills (i.e. active listening, trust-building, etc.)? Did they share characteristics with participants (i.e. veteran status, neighborhood, language, etc.)? What was the supervision structure? Training is described in other related manuscripts in more detail but perhaps 1 sentence could be added here.

- Thank you for these questions about the CHWs. We are cautious about adding too much detail in this paper, given the word limits by the Journal. However, we did add a few more details to the paper as suggested.

- Information on types of Health Action Plans would be informative – did all patients have plans related to diabetes prevention (i.e. weight loss, diet, exercise, etc.)? What were the other types of goals?

- Yes, all Health Action Plans were organized to focus on preventing diabetes, mostly related to goals and plans to lose weight through healthier eating and increased exercise.

- In the discussion, authors note that adherence for the core components of the intervention was high. However, the fidelity manuscript notes that participants had an average of 5 successful encounters throughout the intervention. This seems quite low for an intervention designed to last 12 months with 6 months of weekly contacts (~26) and 6 months of monthly contacts (~6). This is not addressed in the manuscript under review but seems like an important factor limiting effectiveness.

- This is accurate and we now address this as a limitation in the discussion.

- Under limitations, authors describe that “CHW intervention implementation varied” – how so? By implementation site, CHW?

- Although we provided unified training and supervision of CHWs, they were given latitude in this pragmatic trial to do what they thought would be most effective with each patient. Therefore, there was variation in how the protocol was implemented across CHWs and patients.

- In Table 1, the employment category includes just two variables – “self-employed” vs. “others” with over half of participants grouped under “others.” What other categories are included in “others”? It would be worth specifying N and % employed full or part-time, unemployed, disabled, retired, in school, primary caregiver, etc. if data are available.
- Also in Table 1, self-pay/no insurance/unknown are grouped – unknown should be listed as a separate category. I would expect that all participants enrolled at the VA site have VA insurance.

- For the employment category, the others, as the reviewer indicated, included unemployed, disabled, retired, etc., but the categories used by the two sites (BH and VA) were different. That was why we summarize it as two categories.
- For insurance, we understand there are multiple ways to collapse the categorical variable, but to harmonize categories from two sites, this was the most consistent way.
