# Supplementary material for: Community health worker intervention to prevent incident type 2 diabetes: A cluster randomized trial
Source: PLoS One. 2026 Oct 30;21(10):e0361464. doi: 10.1371/journal.pone.0361464 (PMC13722175; doi:10.1371/journal.pone.0361464)
Supplement: Supplementary file 3 [file pone.0361464.s004.docx]

**PLOS ONE Manuscript PONE-D-25-65348R1**
Community Health Worker Intervention to Prevent Incident Type 2 Diabetes: A Cluster Randomized Trial

**Response to Reviewers**

# Reviewer #2 - Comment 1: Study Population

**Reviewer Comment:** The rationale for using a two-year timeframe to identify participants with prediabetes has now been clarified in the manuscript. However, this remains a relatively long interval, during which some participants may have reverted to normoglycemia, while others may have progressed to diabetes. Therefore, it would be appropriate to acknowledge this as a potential limitation of the study in the Discussion section.

**Response:** We agree. Although the two-year window was selected to balance identification of patients with recent prediabetes against misclassification over longer intervals, HbA1c status could have changed before enrollment. We have added this explicitly as a limitation in the Discussion.

**Manuscript Change:** We added the following to the Limitations paragraph in the Discussion: “Eligibility was based on an HbA1c in the prediabetes range within the preceding two years; some participants therefore may have reverted to normoglycemia or progressed to diabetes before enrollment.”

# Reviewer #2 - Comment 2: Diabetes Diagnosis Lookback

**Reviewer Comment:** It's unclear whether the 2-year lookback applies only to the HbA1c inclusion criterion or also to the diabetes diagnosis search. If diagnosis history was also limited to 2 years, patients diagnosed earlier but without a new ICD code or prescription re-recorded in that window could have been missed by the exclusion algorithm. Could the authors clarify how far back the diagnosis check went, or flag this as a limitation if it was indeed restricted to 2 years?

**Response:** Thank you for identifying this issue. The submitted manuscript states that the exclusion algorithm identified ambulatory diabetes diagnosis codes during the preceding two years. We used a two-year lookback search for both diagnostic codes for diabetes and for glycosylated hemoglobin test results. We have stated this explicitly and acknowledged the resulting potential for misclassification.

**Manuscript Change:** We added the following to the Limitations paragraph:
“The algorithm used to exclude prevalent diabetes identified ambulatory diabetes diagnosis codes during the preceding two years; therefore, patients with an earlier diabetes diagnosis that was not recoded during that interval could have been misclassified as not having diabetes.”

# Reviewer #2 - Comment 3: Intervention and Control Conditions

**Reviewer Comment:** I recommend including a more detailed description of the usual care provided to the control group in the Methods section.

**Response:** We agree and have expanded the description of the control condition. Control patients continued to receive usual care from their primary care teams and had access to existing clinical services and programs but received no CHW outreach or other components of the CHORD intervention.

**Manuscript Change:** We added the following to the Methods after the intervention description: “Control patients continued to receive usual care from their primary care teams and had access to existing clinical services and programs but received no CHW outreach or other components of the CHORD intervention.”

# Reviewer #2 - Comment 4: Statistical Analysis

**Reviewer Comment:** Thank you for the clarifications regarding the statistical analyses. I suggest including the version of R used for the analyses, along with the corresponding statistical packages, in the manuscript. Furthermore, I recommend providing a description of the sample size calculation.

**Response:** We agree that additional statistical detail is helpful. We have added the R version used for the analyses (R version 3.4.1) and expanded the description of the sample-size calculation, including the assumed intracluster correlation coefficient. The specific statistical packages used for the original analyses are no longer available to us and therefore cannot be reported reliably.

**Manuscript Change:** We revised the Statistical Analysis section as follows: “Analyses were conducted using R version 3.4.1, with statistical significance set at P<0.05. Sample size was determined to provide at least 80% power to detect a difference in annual diabetes incidence hazard rates of 3.5% in the intervention group versus 5.4% in the control group at a 0.05 significance level, as detailed in the published protocol. The calculation assumed an intracluster correlation coefficient of 0.01.”

# Reviewer #3 - Comment 1: Ethnicity Ascertainment

**Reviewer Comment:** Results: The statement that the intervention group was more likely to be Hispanic or Latino because “the CHWs asked only that question of them” is confusing and somewhat misleading. If accurate, the reviewer recommends clarifying that only intervention participants were asked to self-report ethnicity.

**Response:** Thank you. We agree that the original wording was unclear and have revised the Results to distinguish self-reported ethnicity in the intervention group from ethnicity obtained from the EHR for controls.

**Manuscript Change:** We revised the Results as follows: “Only intervention participants were asked to self-report ethnicity; ethnicity for control participants was obtained from the EHR and was frequently missing. Consequently, a greater proportion of intervention than control participants were identified as Hispanic or Latino.”

# Reviewer #3 - Comment 2: Employment Category

**Reviewer Comment:** In Table 1, the category “Self-employed” typically indicates that the participant works for themself rather than an employer. The reviewer expects that “employed” without the “self-” prefix may be more accurate.

**Response:** Thank you. This was a labeling error. We have corrected “Self Employed” to “Employed” and “Others” to “Other” and clarified the employment categories in the table footnote.

**Manuscript Change:** We revised Table 1 to read “Employed” and “Other” and added the following footnote: “Employment categories harmonized across sites; ‘Other’ includes unemployed, retired, disabled, and other statuses.”

**Reference Review:** In accordance with the journal's request, we reviewed all references for completeness and accuracy and checked cited publications for retractions and corrections. We corrected several bibliographic errors and replaced a withdrawn Cochrane protocol with an active reference. No cited publications were identified as retracted.
