# Supplementary material for: Community health worker intervention to prevent incident type 2 diabetes: A cluster randomized trial
Source: PLoS One. 2026 Oct 30;21(10):e0361464. doi: 10.1371/journal.pone.0361464 (PMC13722175; doi:10.1371/journal.pone.0361464)
Supplement: Peer review history

# Peer review: Decision Letter 0

**DOI:** 10.1371/journal.pone.0361464.r001

**Authors:** Mahmoud Werfalli

19 Mar 2026

Dear Dr. Schwartz,

Please submit your revised manuscript by May 03 2026 11:59PM. If you will need more time than this to complete your revisions, please reply to this message or contact the journal office at plosone@plos.org. When you're ready to submit your revision, log on to https://www.editorialmanager.com/pone/ and select the 'Submissions Needing Revision' folder to locate your manuscript file.

- A letter that responds to each point raised by the academic editor and reviewer(s). You should upload this letter as a separate file labeled 'Response to Reviewers'.
- A marked-up copy of your manuscript that highlights changes made to the original version. You should upload this as a separate file labeled 'Revised Manuscript with Track Changes'.
- An unmarked version of your revised paper without tracked changes. You should upload this as a separate file labeled 'Manuscript'.

If you would like to make changes to your financial disclosure, please include your updated statement in your cover letter. Guidelines for resubmitting your figure files are available below the reviewer comments at the end of this letter.

If applicable, we recommend that you deposit your laboratory protocols in protocols.io to enhance the reproducibility of your results. Protocols.io assigns your protocol its own identifier (DOI) so that it can be cited independently in the future. For instructions see: https://journals.plos.org/plosone/s/submission-guidelines#loc-laboratory-protocols. Additionally, PLOS ONE offers an option for publishing peer-reviewed Lab Protocol articles, which describe protocols hosted on protocols.io. Read more information on sharing protocols at https://plos.org/protocols?utm_medium=editorial-email&utm_source=authorletters&utm_campaign=protocols.

We look forward to receiving your revised manuscript.

Kind regards,

Mahmoud M Werfalli, PhD

Academic Editor

PLOS One

Journal requirements:

When submitting your revision, we need you to address these additional requirements.

1. Please ensure that your manuscript meets PLOS ONE's style requirements, including those for file naming. The PLOS ONE style templates can be found at

https://journals.plos.org/plosone/s/file?id=wjVg/PLOSOne_formatting_sample_main_body.pdf and

https://journals.plos.org/plosone/s/file?id=ba62/PLOSOne_formatting_sample_title_authors_affiliations.pdf

2. Thank you for stating the following financial disclosure:

“This study was supported by a grant (R18DK110740) from the National Institute of Diabetes and Digestive and Kidney Diseases (NIDDK).”

Please state what role the funders took in the study.  If the funders had no role, please state: "The funders had no role in study design, data collection and analysis, decision to publish, or preparation of the manuscript."

If this statement is not correct you must amend it as needed.

Please include this amended Role of Funder statement in your cover letter; we will change the online submission form on your behalf.

3. Thank you for stating the following in your manuscript:

“This study was supported by a grant (R18DK110740) from the National Institute of Diabetes and Digestive and Kidney Diseases (NIDDK). The funder monitored study progress but did not have a role in its design, implementation, analysis, or the preparation of this paper.  “

We note that you have provided funding information that is currently declared in your Funding Statement. However, funding information should not appear in the Acknowledgments section or other areas of your manuscript. We will only publish funding information present in the Funding Statement section of the online submission form.

Please remove any funding-related text from the manuscript and let us know how you would like to update your Funding Statement. Currently, your Funding Statement reads as follows:

“This study was supported by a grant (R18DK110740) from the National Institute of Diabetes and Digestive and Kidney Diseases (NIDDK).”

Please include your amended statements within your cover letter; we will change the online submission form on your behalf.

4. Please provide a complete Data Availability Statement in the submission form, ensuring you include all necessary access information or a reason for why you are unable to make your data freely accessible. If your research concerns only data provided within your submission, please write "All data are in the manuscript and/or supporting information files" as your Data Availability Statement.

Reviewers' comments:

Reviewer's Responses to Questions

**Comments to the Author**

1. Is the manuscript technically sound, and do the data support the conclusions?

Reviewer #1: Yes

Reviewer #2: Partly

Reviewer #3: Yes

**********

2. Has the statistical analysis been performed appropriately and rigorously?

Reviewer #1: No

Reviewer #2: Yes

Reviewer #3: Yes

**********

3. Have the authors made all data underlying the findings in their manuscript fully available?

The PLOS Data policy

Reviewer #1: Yes

Reviewer #2: Yes

Reviewer #3: No

**********

4. Is the manuscript presented in an intelligible fashion and written in standard English?

Reviewer #1: Yes

Reviewer #2: Yes

Reviewer #3: Yes

**********

Reviewer #1: The team conducted a pragmatic, cluster-randomized controlled trial to evaluate whether community health workers intervention can prevent incident T2DM among adults with prediabetes. The results from a 12-month CHW program don’t show significant differences in T2D incident between intervention and control groups.

1. Make sure to spell out the abbreviation for the first appearance. For example, what’s PC?

2. Please clarify how control/intervention PC was determined

3. Many exclusions occurred after randomization, which may lead to biased effect estimates.

4. Sample characteristics at baseline were significantly different but they were not adjusted in the model, which would lead to confounding effect.

5. Please clarify the cluster-randomization”. For example, how many clusters per arm? What’s cluster size and so on.

Reviewer #2: First, I congratulate the authors on this manuscript, which addresses a relevant topic: lifestyle interventions delivered by community health workers hold promise for preventing type 2 diabetes mellitus. The following considerations are offered to further strengthen the work.

Introduction

1. The introduction is clear and direct; however, it would benefit from a more detailed description of diabetes mellitus and its implications, along with greater emphasis on the urgency of intervention in prediabetes.

2. The systematic review in the second paragraph requires additional details, including the number of included studies, the total number of participants, and the geographic regions covered.

3. I recommend rewriting the final sentence of the second paragraph for clarity and supporting it with an appropriate reference.

Methods

4. Although the manuscript references a previously published protocol, it does not provide the registration number or full publication details.

Setting

5. A figure illustrating the content of this section could substantially enhance readers’ understanding of the study.

Study Population

6. The inclusion criterion specifies participants with a history of prediabetes within the last two years; However, the rationale for selecting this timeframe is unclear, as biochemical parameters may change substantially over longer periods.

7. Regarding exclusion criteria, only a diagnosis of diabetes within the last two years is mentioned, despite diabetes being a chronic, incurable condition—individuals diagnosed earlier retain the diagnosis lifelong. Furthermore, the other exclusion criteria require clarification, including rephrasing for precision in the sentence: “PC clinicians in the intervention group reviewed the list of their eligible patients and could exclude any they determined not appropriate for the intervention.”

8. The manuscript lacks essential details on randomization, including who generated the random allocation sequence, the method and type of randomization employed, and any restrictions used. It also omits the mechanism for implementing the sequence (e.g., steps to conceal allocation until interventions were assigned). A dedicated section describing this study stage would improve transparency and reproducibility.

Intervention and Control Conditions

9. The method of contact with participants varied; a subanalysis could assess whether this factor influenced final outcomes. Similarly, participants who sought additional contacts with health agents may have achieved different outcomes than those who received only the scheduled interactions. A figure could more clearly illustrate the intervention logistics. Furthermore, no information is provided regarding the care received (or withheld) from the control group.

Outcome Measures

10. Change in weight in pounds (and body mass index, BMI, kg/m2), glycemic control (HbA1C), blood pressure, and cholesterol levels were secondary outcomes considered, but it was not described how and how frequently these variables were assessed.

Statistical Analysis

11. The manuscript does not clarify whether normality was assessed for continuous variables. Although R software is mentioned for statistical analyses, the specific packages and version employed are not reported. Furthermore, no description of the sample size calculation is provided to justify statistical power.

Results

Study Population

12. The manuscript provides no explanation of how the 1374 participants were selected or the rationale for this specific sample size.

Discussion

13. The first paragraph states that this study did not demonstrate a reduction in diabetes incidence in the intervention group, unlike prior reports. It would be valuable to highlight methodological and intervention differences that may account for this finding.

14. The manuscript omits a discussion of secondary outcomes. Addressing findings from similar recent studies and comparing them to the present results would enrich the work.

15. In the second paragraph, the statement “This may be a positive outcome, as increased engagement with healthcare providers is associated with better management of chronic conditions” lacks a reference. Regarding this statement, it is noteworthy that this study demonstrated a higher rate of comorbidity development (e.g., ischemic heart disease) in the intervention group—the very group that sought health services more frequently. Discussing this finding would also be valuable.

16. In addition to outlining the study's limitations, highlighting its positive aspects is essential.

17. Finally, two key points regarding the study's limitations should be added: differences in baseline characteristics between the two groups and disparities in group sizes (intervention versus control).

Reviewer #3: This interesting paper describes a well-designed large pragmatic trial of a community health worker intervention for pre-diabetic patients. Although the study was ultimately a negative trial, the rigorous yet pragmatic methods for assessing this CHW intervention are an important contribution to the literature. Other strengths include large sample size and appropriate analytic methods. The authors note that the intensity of the intervention may not have been sufficient to demonstrate diabetes prevention; this insight could be explored more by incorporating additional results from the authors’ similarly well-designed manuscript assessing intervention fidelity.

Authors have not included data underlying findings in the manuscript or in a publicly accessible database, but authors describe that it can be made available without restrictions. However, they note that some data currently sit behind VA firewalls, and the VA typically tightly restricts data sharing. Per PLOS One guidance, the authors should be more explicit about the process for obtaining data and any restrictions on data sharing.

The manuscript is well-written and clear. The following minor edits would strengthen clarity:

- Spell out “Veterans Affairs New York” before using VA NY acronym in both abstract and manuscript body.

- On page 9, there is an extraneous parenthesis at the end of the first sentence.

Some additional suggestions that may strengthen this manuscript:

- Additional description of CHWs characteristics, hiring process, training, and supervision would be valuable. How were CHWs identified and hired? Were they selected for specific skills (i.e. active listening, trust-building, etc.)? Did they share characteristics with participants (i.e. veteran status, neighborhood, language, etc.)? What was the supervision structure? Training is described in other related manuscripts in more detail but perhaps 1 sentence could be added here.

- Information on types of Health Action Plans would be informative – did all patients have plans related to diabetes prevention (i.e. weight loss, diet, exercise, etc.)? What were the other types of goals?

- In the discussion, authors note that adherence for the core components of the intervention was high. However, the fidelity manuscript notes that participants had an average of 5 successful encounters throughout the intervention. This seems quite low for an intervention designed to last 12 months with 6 months of weekly contacts (~26) and 6 months of monthly contacts (~6). This is not addressed in the manuscript under review, but seems like an important factor limiting effectiveness.

- Under limitations, authors describe that “CHW intervention implementation varied” – how so? By implementation site, CHW?

- In Table 1, the employment category includes just two variables – “self-employed” vs. “others” with over half of participants grouped under “others.” What other categories are included in “others”? It would be worth specifying N and % employed full or part-time, unemployed, disabled, retired, in school, primary caregiver, etc. if data are available.

- Also in Table 1, self-pay/no insurance/unknown are grouped – unknown should be listed as a separate category. I would expect that all participants enrolled at the VA site have VA insurance.

**********

what does this mean?). If published, this will include your full peer review and any attached files.

If you choose “no”, your identity will remain anonymous but your review may still be made public.

**Do you want your identity to be public for this peer review?**  For information about this choice, including consent withdrawal, please see our Privacy Policy

Reviewer #1: No

Reviewer #2: No

Reviewer #3: No

**********

[NOTE: If reviewer comments were submitted as an attachment file, they will be attached to this email and accessible via the submission site. Please log into your account, locate the manuscript record, and check for the action link "View Attachments". If this link does not appear, there are no attachment files.]

To ensure your figures meet our technical requirements, please review our figure guidelines: https://journals.plos.org/plosone/s/figures

You may also use PLOS’s free figure tool, NAAS, to help you prepare publication quality figures: https://journals.plos.org/plosone/s/figures#loc-tools-for-figure-preparation.

NAAS will assess whether your figures meet our technical requirements by comparing each figure against our figure specifications.

# Author comment: Author response to Decision Letter 1

**DOI:** 10.1371/journal.pone.0361464.r002

22 May 2026

Please see the uploaded "Response to Editorial Review"

Attachment Submitted filename: Response to Editorial Review.docx

# Peer review: Decision Letter 1

**DOI:** 10.1371/journal.pone.0361464.r003

**Authors:** Katrien Janin

1 Sep 2026

Dear Dr. Schwartz,

Thank you for submitting your manuscript to PLOS One. After careful consideration, we feel that it has merit but does not fully meet PLOS One’s publication criteria as it currently stands. Therefore, we invite you to submit a revised version of the manuscript that addresses the points raised during the review process.

Please submit your revised manuscript by Oct 16 2026 11:59PM. If you will need more time than this to complete your revisions, please reply to this message or contact the journal office at plosone@plos.org. When you're ready to submit your revision, log on to https://www.editorialmanager.com/pone/ and select the 'Submissions Needing Revision' folder to locate your manuscript file.

- A letter that responds to each point raised by the academic editor and reviewer(s). You should upload this letter as a separate file labeled 'Response to Reviewers'.
- A marked-up copy of your manuscript that highlights changes made to the original version. You should upload this as a separate file labeled 'Revised Manuscript with Track Changes'.
- An unmarked version of your revised paper without tracked changes. You should upload this as a separate file labeled 'Manuscript'.

If you would like to make changes to your financial disclosure, please include your updated statement in your cover letter. Guidelines for resubmitting your figure files are available below the reviewer comments at the end of this letter.

If applicable, we recommend that you deposit your laboratory protocols in protocols.io to enhance the reproducibility of your results. Protocols.io assigns your protocol its own identifier (DOI) so that it can be cited independently in the future. For instructions see: https://journals.plos.org/plosone/s/submission-guidelines#loc-laboratory-protocols. Additionally, PLOS One offers an option for publishing peer-reviewed Lab Protocol articles, which describe protocols hosted on protocols.io. Read more information on sharing protocols at https://plos.org/protocols?utm_medium=editorial-email&utm_source=authorletters&utm_campaign=protocols.

As the corresponding author, your ORCID iD is verified in the submission system and will appear in the published article. PLOS supports the use of ORCID, and we encourage all coauthors to register for an ORCID iD and use it as well. Please encourage your coauthors to verify their ORCID iD within the submission system before final acceptance, as unverified ORCID iDs will not appear in the published article. *Only*  the individual author can complete the verification step; PLOS staff *cannot*  verify ORCID iDs on behalf of authors.

We look forward to receiving your revised manuscript.

Kind regards,

Katrien G. Janin, PhD

Staff Editor

PLOS One

Journal Requirements:

If the reviewer comments include a recommendation to cite specific previously published works, please review and evaluate these publications to determine whether they are relevant and should be cited. There is no requirement to cite these works unless the editor has indicated otherwise.

Please review your reference list to ensure that it is complete and correct. If you have cited papers that have been retracted, please include the rationale for doing so in the manuscript text, or remove these references and replace them with relevant current references. Any changes to the reference list should be mentioned in the rebuttal letter that accompanies your revised manuscript. If you need to cite a retracted article, indicate the article’s retracted status in the References list and also include a citation and full reference for the retraction notice.

[Note: HTML markup is below. Please do not edit.]

Reviewers' comments:

Reviewer's Responses to Questions

**Comments to the Author**

Reviewer #1: All comments have been addressed

Reviewer #2: (No Response)

Reviewer #3: (No Response)

**********

2. Is the manuscript technically sound, and do the data support the conclusions?

Reviewer #1: (No Response)

Reviewer #2: Yes

Reviewer #3: Yes

**********

3. Has the statistical analysis been performed appropriately and rigorously?

Reviewer #1: (No Response)

Reviewer #2: Yes

Reviewer #3: Yes

**********

4. Have the authors made all data underlying the findings in their manuscript fully available?

The PLOS Data policy

Reviewer #1: (No Response)

Reviewer #2: Yes

Reviewer #3: Yes

**********

5. Is the manuscript presented in an intelligible fashion and written in standard English?

Reviewer #1: (No Response)

Reviewer #2: Yes

Reviewer #3: Yes

**********

Reviewer #1: (No Response)

Reviewer #2: Thank you for the clarifications provided. Below are a few points that would benefit from further clarification:

Study Population

1. The rationale for using a two-year timeframe to identify participants with prediabetes has now been clarified in the manuscript. However, this remains a relatively long interval, during which some participants may have reverted to normoglycemia, while others may have progressed to diabetes. Therefore, it would be appropriate to acknowledge this as a potential limitation of the study in the Discussion section.

2. It's unclear whether the 2-year lookback applies only to the HbA1c inclusion criterion or also to the diabetes diagnosis search. If diagnosis history was also limited to 2 years, patients diagnosed earlier but without a new ICD code or prescription re-recorded in that window could have been missed by the exclusion algorithm — this can happen when chronic diagnoses sit on a static problem list without being re-coded at each visit.

The HbA1c criterion likely reduces this risk, since most treated diabetics would fall outside the 5.7–6.4% range, but it doesn't fully rule out cases of remission or tight glycemic control. Could the authors clarify how far back the diagnosis check went, or flag this as a limitation if it was indeed restricted to 2 years?

Intervention and Control Conditions

3. I recommend including a more detailed description of the usual care provided to the control group in the Methods section.

Statistical Analysis

4. Thank you for the clarifications regarding the statistical analyses. I suggest including the version of R used for the analyses, along with the corresponding statistical packages, in the manuscript. Furthermore, I recommend providing a description of the sample size calculation.

Reviewer #3: The authors' revisions have greatly strengthened the manuscript. Two minor suggestions:

Results: On page 14, the authors' revised manuscript now notes that "...the intervention group was more likely to be Hispanic or Latino, as the CHWs asked only that question of them." This is confusing and somewhat misleading, since it sounds like the control group was not given an opportunity to report ethnicity and that it was otherwise abstracted from EHR which had substantial missing data. I recommend instead (if accurate) "Only intervention group participants were asked to self-report ethnicity, and therefore more intervention participants identified as Hispanic or Latino compared with controls."

In the table, authors clarify that incomplete and inconsistent data was available about employment, resulting in "self-employed vs. other" categories. "Self-employed" typically indicates that the participant has their own business and works for themselves, not an employer - I expect that "employed" without the "self-" prefix is more accurate.

**********

what does this mean?). If published, this will include your full peer review and any attached files.

If you choose “no”, your identity will remain anonymous but your review may still be made public.

**Do you want your identity to be public for this peer review?**  For information about this choice, including consent withdrawal, please see our Privacy Policy

Reviewer #1: No

Reviewer #2: No

Reviewer #3: No

**********

[NOTE: If reviewer comments were submitted as an attachment file, they will be attached to this email and accessible via the submission site. Please log into your account, locate the manuscript record, and check for the action link "View Attachments". If this link does not appear, there are no attachment files.]

To ensure your figures meet our technical requirements, please review our figure guidelines: https://journals.plos.org/plosone/s/figures

You may also use PLOS’s free figure tool, NAAS, to help you prepare publication quality figures: https://journals.plos.org/plosone/s/figures#loc-tools-for-figure-preparation.

NAAS will assess whether your figures meet our technical requirements by comparing each figure against our figure specifications.

# Author comment: Author response to Decision Letter 2

**DOI:** 10.1371/journal.pone.0361464.r004

29 Sep 2026

Dr. Janin,

As we describe in the enclosed cover letter, we greatly appreciate the additional feedback and the opportunity to respond and improve this paper. We look forward to your review and decision.

Best,

Mark D Schwartz MD

Attachment Submitted filename: Response to Reviewers PLOS One 2026-9-27.docx

# Peer review: Decision Letter 2

**DOI:** 10.1371/journal.pone.0361464.r005

**Authors:** Sana Sheikh

12 Oct 2026

Community Health Worker Intervention to Prevent Incident Type 2 Diabetes: A Cluster Randomized Trial

PONE-D-25-65348R2

Dear Dr. Schwartz,

We’re pleased to inform you that your manuscript has been judged scientifically suitable for publication and will be formally accepted for publication once it meets all outstanding technical requirements.

Within one week, you’ll receive an e-mail detailing the required amendments. When these have been addressed, you’ll receive a formal acceptance letter and your manuscript will be scheduled for publication.

An invoice will be generated when your article is formally accepted. Please note, if your institution has a publishing partnership with PLOS and your article meets the relevant criteria, all or part of your publication costs will be covered. Please make sure your user information is up-to-date by logging into Editorial Manager at Editorial Manager® and clicking the ‘Update My Information' link at the top of the page. For questions related to billing, please contact billing support.

If your institution or institutions have a press office, please notify them about your upcoming paper to help maximize its impact. If they’ll be preparing press materials, please inform our press team as soon as possible -- no later than 48 hours after receiving the formal acceptance. Your manuscript will remain under strict press embargo until 2 pm Eastern Time on the date of publication. For more information, please contact onepress@plos.org.

Kind regards,

Sana Sadiq Sheikh

Academic Editor

PLOS One

Additional Editor Comments (optional):

Reviewers' comments:

Reviewer's Responses to Questions

**Comments to the Author**

Reviewer #1: All comments have been addressed

Reviewer #2: All comments have been addressed

Reviewer #3: All comments have been addressed

**********

2. Is the manuscript technically sound, and do the data support the conclusions?

Reviewer #1: (No Response)

Reviewer #2: Yes

Reviewer #3: Yes

**********

3. Has the statistical analysis been performed appropriately and rigorously?

Reviewer #1: (No Response)

Reviewer #2: Yes

Reviewer #3: Yes

**********

4. Have the authors made all data underlying the findings in their manuscript fully available?

The PLOS Data policy

Reviewer #1: (No Response)

Reviewer #2: Yes

Reviewer #3: Yes

**********

5. Is the manuscript presented in an intelligible fashion and written in standard English?

Reviewer #1: (No Response)

Reviewer #2: Yes

Reviewer #3: Yes

**********

Reviewer #1: (No Response)

Reviewer #2: (No Response)

Reviewer #3: The authors have adequately addressed all reviewer comments in this revision. Thank you for the opportunity to review this manuscript.

**********

what does this mean?). If published, this will include your full peer review and any attached files.

If you choose “no”, your identity will remain anonymous but your review may still be made public.

**Do you want your identity to be public for this peer review?**  For information about this choice, including consent withdrawal, please see our Privacy Policy

Reviewer #1: No

Reviewer #2: No

Reviewer #3: No

**********

# Editor report: Acceptance letter

**DOI:** 10.1371/journal.pone.0361464.r006

**Authors:** Sana Sheikh

PONE-D-25-65348R2

PLOS One

Dear Dr. Schwartz,

I'm pleased to inform you that your manuscript has been deemed suitable for publication in PLOS One. Congratulations! Your manuscript is now being handed over to our production team.

At this stage, our production department will prepare your paper for publication. This includes ensuring the following:

* All references, tables, and figures are properly cited

* All relevant supporting information is included in the manuscript submission,

* There are no issues that prevent the paper from being properly typeset

You will receive further instructions from the production team, including instructions on how to review your proof when it is ready. Please keep in mind that we are working through a large volume of accepted articles, so please give us a few days to review your paper and let you know the next and final steps.

Lastly, if your institution or institutions have a press office, please let them know about your upcoming paper now to help maximize its impact. If they'll be preparing press materials, please inform our press team within the next 48 hours. Your manuscript will remain under strict press embargo until 2 pm Eastern Time on the date of publication. For more information, please contact onepress@plos.org.

You will receive an invoice from PLOS for your publication fee after your manuscript has reached the completed accept phase. If you receive an email requesting payment before acceptance or for any other service, this may be a phishing scheme. Learn how to identify phishing emails and protect your accounts at https://explore.plos.org/phishing.

If we can help with anything else, please email us at customercare@plos.org.

Thank you for submitting your work to PLOS One and supporting open access.

Kind regards,

PLOS One Editorial Office Staff

on behalf of

Dr. Sana Sadiq Sheikh

Academic Editor

PLOS One
